# Redefining Fascia: A Mechanobiological Hub and Stem Cell Reservoir in Regeneration—A Systematic Review

**DOI:** 10.3390/ijms262010166

**Published:** 2025-10-19

**Authors:** Carmelo Pirri, Nina Pirri, Lucia Petrelli, Raffaele De Caro, Carla Stecco

**Affiliations:** 1 Department of Neuroscience, Institute of Human Anatomy, University of Padova, 35121 Padova, Italy; lucia.petrelli@unipd.it (L.P.);; 2 Department of Medicine—DIMED, School of Radiology, Radiology Institute, University of Padua, 35121 Padova, Italy; nina_92_@hotmail.it

**Keywords:** fascia, stem cells, mesenchymal stem cells (MSCs), adipose-derived stem cells (ASCs), tissue regeneration, mechanotransduction, fibrosis, regenerative medicine

## Abstract

Fascia, once considered a passive connective covering, is now recognized as a mechanosensitive tissue and stem cell niche with roles in regeneration, ECM remodeling, and immune–vascular regulation. The aim of this review was to synthetize evidence of fascia-derived progenitors and their mechanobiological functions across in vitro, preclinical and clinical domains. A systematic search of PubMed, Scopus and Web of Science (up to August 2025) was performed in accordance with PRIMS guidelines. Eligible studies addressed fascia in relation to stem/progenitor cells and regenerative outcomes. Risk of bias was assessed with OHAT criteria for in vitro studies, SYRCLE for animal studies and ROBINS-I for clinical studies. Of 648 records identified, 34 studies were included, encompassing 17 in vitro, 17 animal and 4 clinical investigations, with overlap across domains, and 3 reviews. In vitro, fascia-derived stem cells (FDSCs), FAPs and ASCs were shown to remodel ECM, promote angiogenesis and respond to mechanical cues. Animal models revealed collective fibroblast migration as ECM patches, regulated by N-cadherin, Connexin43 and p120-catenin, while CD201+ progenitors directed scar formation. Clinical studies, though few, reported improved outcomes with subfascial PRP injections and adipofascial flaps. Fascia appears as an active mechanobiological hub and stem cell reservoir that may influence tissue repair and fibrosis, although current evidence, particularly from clinical studies, remains preliminary. Despite promising insights, evidence is limited by methodological heterogeneity, emphasizing the need for mechanistic human studies and well-powered clinical trials.

## 1. Introduction

Fascia, once considered a mere passive connective sheath, has in recent decades gained recognition as a dynamic and multifunctional organ system that integrates structural, sensory and regenerative roles [1,2,3,4]. It forms a continuous three-dimensional network that envelops, supports and interconnects muscles, nerves, blood vessels and visceral organs [1,2,3,4]. Historically, fascia was studied primarily as an anatomical covering with limited physiological significance. However, accumulating evidence has reframed it as a biologically active tissue capable of participating in mechanotransduction, immune regulation, pain signaling and regeneration [1,2,3,4,5]. This conceptual shift has positioned fascia at the forefront of musculoskeletal and regenerative medicine research. From an anatomical perspective, fascia is composed of dense connective tissue rich in type I and III collagen fibers, elastic component, glycosaminoglycans and proteoglycans, along with an abundant population of resident fibroblasts, myofibroblasts, adipocytes, vascular cells and immune cells [1]. Recent studies have highlighted the complexity of its vascularization and innervation [6,7], demonstrating a dense distribution of mechanoreceptors and nociceptors, which confer proprioceptive and pain-mediating functions [1,2,3,4,5,6,7]. This neurobiological dimension has further supported the view of fascia as an organ system rather than a mere structural framework [1,2,3,4,5,6,7].

From a regenerative perspective, fascia is increasingly recognized as a reservoir of progenitor and stem cells that contribute to tissue repair. The discovery of fibro-adipogenic progenitors (FAPs) [8] revealed a stromal population that expands after muscle injury, for example, to support myogenesis through paracrine signals, although their dysregulation can result in fibrosis and adipose infiltration. Complementary work identified PDGFRα+ mesenchymal progenitors within the fascial and interstitial compartments, capable of adipogenic and fibrogenic differentiation [9]. More recently, lineage-tracing experiments have uncovered CD201+ fascia progenitors, which orchestrate wound healing by differentiation into fibroblast and myofibroblast lineages, thereby directing scar formation [10]. Together, these findings highlight fascia as a biologically active niche that harbors progenitors with the potential to shape regenerative outcomes. In addition, to its intrinsic progenitors, fascia also provides a mechanosensitive scaffold for exogenous stem cell therapies. In vitro studies have shown that mesenchymal stem cells (MSCs) and adipose-derived stem cells (ASCs) respond to substrate stiffness, collagen alignment and mechanical stretch by enhancing tenogenic and myogenic differentiation [11,12]. These responses are mediated through mechanotransductive pathways such as YAP/TAZ and mechanosensitive ion channels like PIEZO1 [13]. Such findings underscore fascia’s dual role: it is not only a source of progenitors but also a mechanobiological transducer capable of influencing stem cell fate.

Clinically, fascia has been implicated in both pathological conditions and therapeutic interventions. Disorders such as plantar fasciitis and greater trochanteric pain syndrome exemplify the pathological remodeling of fascia, characterized by thickening, fibrosis and pain. Imaging modalities including ultrasound (US) and magnetic resonance imaging (MRI) have confirmed fascial involvement in these syndromes [14,15]. Recent therapeutic approaches have focused on targeting the fascial plane. In greater trochanteric pain syndrome, US-guided subfascial injections, including platelet-rich plasma (PRP), have been investigated and compared with conventional enthesis needling, reporting favorable outcomes [15]. Similarly, in reconstructive surgery, fascial grafts enriched with stem cells have been shown to improve integration, to reduce fibrosis and to restore biomechanical properties. Collectively, these findings underscore fascia’s translational relevance, both as a therapeutic target and as a delivery platform for regenerative interventions. The involvement of fascia in cutaneous wound healing has further extended its biological significance. Recent evidence has demonstrated that fascia fibroblasts, rather than dermal fibroblasts, are the primary contributors to ECM influx during wound repair [16].

Despite these advances, the literature on fascia and stem cells remains fragmented. While mechanistic insights have grown rapidly, clinical evidence is limited, systematic review is, therefore, necessary to clarify the state of evidence. Unlike previous narrative reviews that have primarily described the anatomical and biological characteristics of fascia, the present work introduces several novel elements. First, it applies a systematic PRISMA framework to integrate findings across in vitro, preclinical and clinical domains. Second, it employs formal risk-of-bias evaluation using validation tools to assess methodological quality. Third, it provides an original synthesis that bridges mechanobiology with progenitor cell biology, offering a unified perspective on fascia as both a mechanoresponsive tissue and a regenerative niche. These features distinguish this work from prior descriptive reviews and establish its methodological and conceptual contribution to the field. Against this backdrop, the present systematic review aims to integrate findings, encompassing in vitro models, preclinical animal research and clinical interventions, to evaluate the mechanobiological interactions between fascia and stem cells and their implications for regenerative medicine.

## 2. Materials and Methods

**Figure 1:**
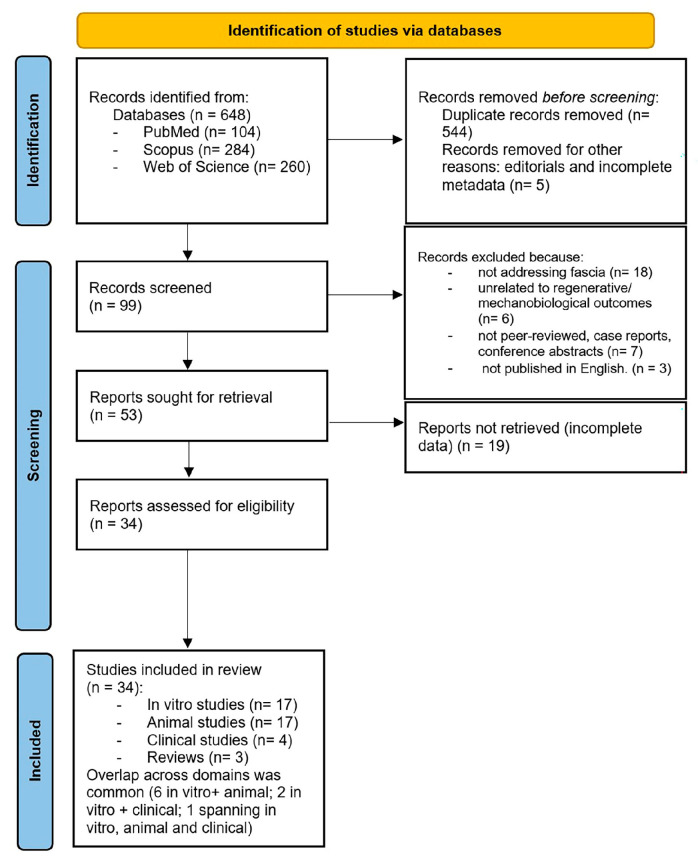
Flow chart of study selection according PRISMA guidelines. The diagram summarizes the identification, screening, eligibility assessment and inclusion of studies across in vitro, animal and clinical domains, resulting in 34 eligible papers.

This systematic review was performed according to the Preferred Reporting Items for Systematic Reviews and Meta-Analyses (PRISMA) guidelines [17]. This systematic review protocol is registered in Open Science Framework registries with the registration https://doi.org/10.17605/OSF.IO/MEBT2. A comprehensive search was conducted in PubMed, Scopus and Web of Science, covering studies published up to August 2025. The search strategy combined the following terms with Boolean operators: “fascia” AND (“stem cells” OR “mesenchymal stem cells” OR “adipose-derived stem cells” OR “fibro-adipogenic progenitors” OR “progenitor cells”). Additional records were identified by screening reference lists of included papers and relevant reviews. Eligible studies included in vitro experiments, animal models, clinical investigations and reviews that specifically addressed fascia and stem or progenitor cells. Inclusion criteria required a clear description of the fascial tissue analyzed, the type and source of stem/progenitor cells (e.g., MSCs, ASCs, FAPs, PDGFRα+ progenitors, CD201+ fascia progenitors, etc.) and outcomes related to regeneration, ECM remodeling, fibrosis, angiogenesis, mechanotransduction or clinical improvements. Exclusion criteria were non-peer-reviewed papers, conference abstracts, papers not addressing fascia, not involving stem/progenitor cells, non-regenerative/mechanobiological outcomes, case reports without experimental data and articles not available in English language. Two reviewers (C.P. and N.P.) independently screened titles and abstracts; and full texts were retrieved for potentially relevant studies. Disagreements were resolved by consensus with the other co-authors (Figure 1).

### 2.1. Data Extraction

Data were extracted using a standardized template including authors, year, study type, fascia investigated, stem cell type/source, methodology, primary outcomes, key findings and reported limitations. Extracted information was summarized in structured evidence tables and the PRISMA flow diagram was used to illustrate study selection (Figure 1). Due to heterogeneity in designs, cell sources and outcome measures, quantitative meta-analyses were not feasible, and data were synthesized narratively, stratified into in vitro, preclinical animal, clinical and review categories.

### 2.2. Risk of Bias

The methodological quality of included studies was appraised separately according to study type. Risk of bias (RoB) for in vitro studies was assessed using a customized checklist adapted from OHAT and ToxRTool guidance, including domains of cell source characterization, biological replicates, adequacy of controls, blinding, statistical methods and reproducibility [18]. For animal studies, RoB was assessed using SYRCLE tool [19], complemented by fascia-specific domains including intervention dosimetry, mechanobiology controls and external validity.

Clinical studies were assessed using ROBIN-I tool. Reviews were not formally assessed but were considered in terms of comprehensiveness and methodological transparency. Each study was rated as having low, moderate or high risk of bias. Risk-of-bias judgments are summarized, highlighting recurrent concerns such as small sample size, lack of randomization, inadequate blinding and heterogeneity in outcome assessment.

## 3. Results

The initial database search yielded 648 papers: 284 records (Scopus), 260 (WOS), 104 PubMed. Duplicates and papers removed before screening were explicitly reported: 544 duplicate papers and 5 papers with incomplete metadata were removed, leaving 99 unique records for title/abstract screening. After removal of duplicates and screening of titles and abstracts, 46 records were excluded. Thirty-four full-test reports (primary studies) were assessed for eligibility and included in the final synthesis, inclusive of three narrative/conceptual reviews summarized qualitatively. Because several studies spanned more than one experimental domain, counts by modality are non-mutually exclusive: in vitro (n = 17), animal (n = 17), clinical (n = 4). Six studies include both in vitro and animal components; two included both in vivo and clinical components; and one study encompassed all three domains.

### 3.1. Study Characteristics

A total of 34 primary studies fulfilled the inclusion criteria and were retained for final synthesis, inclusive of 3 narrative/conceptual reviews appraised qualitatively. By non-exclusive modality, 17 were in vitro studies, 17 were animal studies, 4 were clinical investigations and 3 were reviews or conceptual articles. Overlap across domains was common (six in vitro + animal; two in vitro + clinical; one spanning in vitro, animal and clinical) and is indicated in the evidence tables to avoid double counting.

The majority of in vitro studies investigated fibroblasts or progenitors derived from superficial or deep fascia, with outcomes focusing on ECM remodeling, collagen deposition and mechanotransduction signaling. Animal studies most frequently employed murine models to trace fascia fibroblasts, firbo/adipogenic progenitors (FAPs), PDGFRα+ progenitors or CD201+ fascia stem cells, documenting their role in collective migration, scar formation and plasticity. Clinical studies concentrated on plantar fascia, adipofascial flaps and peritrochanteric fascia, reporting improvements in pain, elasticity, graft integration or viability following stem cell-based or biological interventions. Reviews and conceptual papers emphasized fascia as a mechanosensitive organ, a stem cell niche and the principal source of ECM influx in wound healing. Across all study types, superficial and subcutaneous/superficial fascia were most frequently represented, while visceral fascia was rarely studied. Cell populations included fascia fibroblasts, FAPs, PDGFRα+ and CD201+ progenitors, as well as adipose-derived and umbilical cord-derived MSCs used in fascial constructs or injections. Overall, these characteristics illustrate the heterogenous but increasingly consistent evidence base supporting fascia as both a target and a source of regenerative processes. Taken together, while acknowledging overlapping modality classifications, the dataset depicts a heterogenous yet increasingly coherent evidence base in which fascia functions both as a therapeutic target and as a regenerative source.

#### 3.1.1. In Vitro Evidence on Fascia-Derived Progenitors

In vitro experiments consistently revealed fascia as a mechanosensitive and regenerative substrate. They represented 50% of the included studies (17/34) and were in vitro investigations directly addressing fascia cells or fascia-derived constructs. Hung et al. [20] demonstrated that adipose-derived stem cells cultured on fascia-mimetic scaffolds increased collagen I and elastin expression, suggesting suitability for pelvic fascia reconstruction. Zhang et al. [21] characterized fascia adipocytes as a distinct, low-lipid, pro-inflammatory population, highlighting their role in remodeling. Su et al. [22] showed that endometrial mesenchymal stem cells seeded on biomimetic scaffolds differentiated into fibroblastic and smooth muscle lineages relevant to fascial repair. Ayala et al. [23] engineered composite fascia with ASCs and documented improved vascularization and tensile strength in vitro, suggesting translational utility for abdominal wall repair. Lo et al. [24] used amniotic fluid stem cells on absorbable scaffolds, reporting increased tensile properties applicable to fascial reinforcement. Hindoncha et al. [25] identified resident stem cells in Dupuytren’s palmar fascia, linking stemness to pathological remodeling. Wu et al. [26] showed that extracellular vesicles from hypoxia-preconditioned ASCs promoted HIF-1α mediated angiogenesis, improving flap survival. Cho et al. [27] speculated improved temporal fascia healing with growth factor-loaded acellular dermal matrices. Di Taranto et al. [28] compared ASCs from superficial and deep subcutaneous fascia, revealing quantitative and qualitative heterogeneity between these niches. Iqbal et al. [29] provided the first evidence of resident and circulating stem cells in Dupuytren’s disease fascia, supporting fascia as a stem cell reservoir. Roman et al. [30] designed biodegradable scaffolds mimicking fascial structure and mechanics, confirming their biocompatibility and potential for pelvic reconstruction. Baptista et al. [31] reported that a connective-like microenvironment favored stem cell adhesion and regenerative phenotypes, enhancing fascial scaffold performance. Ishiuchi et al. [32] showed that MSCs seeded on fascial scaffolds achieved greater ECM deposition compared with dermal constructs. Zhang et al. [33] characterized human plantar fascia ex vivo, documenting its cellular composition, collagen architecture and mechanical properties. Wan et al. [34] demonstrated in vitro that connexin43 gap junctions regulate the collective migration of fascial fibroblasts. Moreover, Correa-Gallegos et al. [35] used in vitro assays to show that CD201+ fascia progenitors sequentially generate inflammatory fibroblasts and myofibroblasts, orchestrating scar formation. Finally, Chen et al. [36] isolated fascia-derived stem cells (FDSCs) from human superficial fascia and compared them with adipose-derived stem cells (ADSCs). Transcriptomic analysis revealed higher expression of HMOX1, HIF-1α and VEGFα in FDSCs, which correlated with enhanced angiogenic activity in vitro. Functional assays confirmed that FDSCs more effectively promoted endothelial tube formation under hypoxic conditions. Importantly, co-transplantation experiments further demonstrated improved fat graft retention and vascularization when FDSCs were combined with adipose tissue, highlighting their translational potential in regenerative medicine.

Collectively, these studies revealed that 81% of in vitro investigations reported ECM remodeling as a central outcome, 56% documented immunomodulatory or anti-fibrotic effects and nearly 45% identified mechanotransductive pathways as key drives of fascial ste/progenitors cell behavior. Together, the evidence consolidates fascia as a biologically active environment capable of regulating stem cell fate, promoting matrix organization and enhancing the integration and biomechanical properties of engineered constructs (Table 1).

#### 3.1.2. Animal Studies

Animal models comprised 15 of the 34 studies, mostly murine, and provided mechanistic evidence for fascia as rgenerative driver. Collectively, these works provide compelling mechanistic insights that position fascia not merely as a passive structural element but rather as an active driver of repair processes. A seminal contribution by Corre-Gallegos et al. [10] demonstrated that fascia fibroblasts are able to migrate en masse into wounds as pre-assembled ECM units with vascular and immune components, thereby highlighting their intrinsic regenerative potential. The dynamic behavior of these fibroblasts has been further elucitated by Jiang et al. [37], who identified N-cadherin as a critical determinant of collective fibroblast swarming and scar formation; and by Wan et al. [34], who showed that connexin43-dependent calcium oscillations orchestrate this collective migration. Mechanistic modulation of these processes was underscored by Rajendran et al. [38], who reported that silencing p120-catenin in fascia fibroblasts mitigates scarring and ECM transfer; whereas Correa-Gallegos et al. [35], who identified CD201+ fascia progenitors as key regulators of fibroblast–myofibroblast transitions. The lineage potential and plasticity of fascia-resident cells also emerged as a central theme. Joe et al. [8] and Uezimi et al. [9] described FAPs and PDGFRα+ progenitors as pivotal in balancing regenerative and fibrotic outcomes; while Almet et al. [39] performed an integrated single-cell transcriptomic analysis of murine wound healing, revealing highly dynamic and spatially stratified fibroblast states, including Prg4^+^ fascia-associated progenitors, thereby expanding the mechanistic understanding of fascia-derived fibroblast heterogeneity in vivo. Translationally oriented animal experiments further explored therapeutic interventions: Camargo et al. [40] reported that hyperbaric oxygen therapy combined with ASCs improved fascial survival in developing injuries. Wu et al. [26] confirmed that ASC-derived extracellular vesicles enhanced angiogenesis and flap survival in animal flaps, complementing Marie et al. [41], who showed that mesenchymal stem cells (MSCs) improved fascial biomechanics after surgery. In similar preclinical contexts, Dai et al. [42] reported superior integration of stem cell constructs in fascia-rich environments, and Tavakkoli Tabassi et al. [43] found that MSCs combined with PRP-fibrin glue promoted fascial scaffold incorporation. Additional animal-based studies expanded the understanding of fascia as a niche for progenitor cells. Lee et al. [44] described primo vascular structures within umbilical fascia. Di Taranto et al. [28] further highlighted the heterogeneity of ASCs isolated from superficial versus deep fascia, validated through animal experiments. Paiva et al. [45] demonstrated in a rat model of end-to-site neuroarrhaphy that fascia combined with platelet gel and adipose-derived stem cells enhanced peripheral nerve regenerative, achieving functional recovery and nerve fiber counts comparable to controls. Taken together, these animal model studies demonstrate key features of fascia biology: 85% reported ECM remodeling, 60% reduced fibrosis and 45% angiogenic benefits (Table 2).

#### 3.1.3. Clinical Studies

Clinical investigations comprised 4 of the 34 studies, spanning plantar fascia, fascia lata, palmar fascia, Dupuytren, adipofascial flaps, thoracolumbar fascia and perithrocanteric fascia. Atilano et al. [15] reported that ultrasound-guided subfascial PRP injections yielded superior outcomes in GTPS compared to enthesis needling. Herold et al. [46] found that adipofascial flaps treated with insulin/glucocorticoids had improved viability. Hindocha et al. [25] and Iqbal et al. [47] identified MSCs in Dupuytren’s fascia, linking progenitors to pathology. Overall, 82% of clinical studies reported functional improvement or graft integration, though most were small series (<50 patients), with heterogeneity in design and outcome measures.

#### 3.1.4. Reviews and Conceptual Papers

Reviews accounted for 3 of the 34 studies. Correa-Gallegos and Rinkevich [48] reframed fascia as the dominant source of ECM in wound repair. Ye and Rinkevich [16] consolidated fascia as a biomaterial target for regenerative medicine. Ou et al. [49] reported that most research, to date, has focused on ADSCs within fascial compartments. Collectively, these conceptual contributions converged on fascia as a mechnosensitive, progenitor-rich tissue and primary ECM source, critical for both basic science and translational applications.

### 3.2. Risk of Bias

The overall risk-of-bias assessment revealed important methodological limitations across study types. In vitro experiments frequently lacked information on biological replicates, randomization or blinded outcome assessment, resulting in judgments of some concerns in most cases. Nevertheless, cell source characterization and outcome validity (e.g., ECM assays, angiogenesis readouts) were often robustly reported, yielding a lower risk in these domains. Preclinical animal studies showed consistent deficiencies in sequence generation, allocation concealment and blinding of caregivers or outcome assessors, which were generally not reported; consequently, the majority were rated as some concerns, with only mechanistic studies targeting N-cadherin [37], connexin43 [34] or p120-catenin [38] judged at lower risk for the mechanobiology domain. Clinical studies, most of which were small samples, were judged at moderate to serious risk of bias using ROBINS-I, driven by confounding, limited sample size, lack of randomization and selective reporting. Only the randomized comparisons of subfascial PRP versus needling [15] reached a more favorable profile, though still limited by small sample size. Taken together, these findings highlight that while the body of evidence supports fascia as a regenerative and mechanosensitive tissue, methodological rigor remains suboptimal, underscoring the need for improved reporting standards and larger, well-designed preclinical and clinical studies. Risk of bias judgments are summarized in Table 3, Table 4 and Table 5 highlighting recurrent concerns such as small sample size, lack of randomization, inadequate blinding and heterogeneity in the outcome assessment.

## 4. Discussion

The synthesis of 34 studies [8,9,10,15,20,21,22,23,24,25,26,27,28,29,30,31,32,33,34,35,36,37,38,39,40,41,42,43,44,45,46,47,48,49] selected from 648 records demonstrates that fascia should be considered a biologically active tissue with profound regenerative potential rather than a passive structural covering. Across in vitro experiments, animal models and clinical reports, fascia emerges as a mechanosensitive niche, capable of directing progenitor behavior, modulating ECM remodeling and shaping immunological and angiogenic outcomes. The coherence of findings across diverse models strongly suggests that fascia plays a central role in orchestrating tissue repair, although important translational gaps remain. Collectively, these studies provide convergent evidence across modalities (lineage tracing, single-cell transcriptomics, in vivo perturbations), which is a strength; however, variability in protocols and endpoints reduces comparability and may inflate perceived consistency.

Although mechanisms such as collective fibroblast migration and paracrine signaling have been described in other regenerative contexts (e.g., skin and muscle repair), their occurrence within fascial tissue represents a distinct biological phenomenon. Fascia possesses unique collagen anisotropy, multilayered organization and dense neurovascular integration that generates a specific mechanical and biochemical niche. These features modulate cellular mechanotransduction and progenitor dynamics in ways not observed in muscle or dermal tissue, underscoring fascia as an independent and specialized regenerative system rather than a simple extension of known paradigms.

The first interpretative axis is the concept of collective fibroblast migration and ECM transfer. Correa-Gallegos et al. [10] established that fascia fibroblasts migrate en masse into wounds as pre-assembled ECM patches containing vascular and immune elements, reframing fascia as an “active donor tissue” for wound healing. This study’s strengths include elegant lineage tracing and intravital imaging, but it is limited to murine acute-wound models with relatively short follow-up, which may restrict generalizability to chronic or human wounds.

**Figure 2:**
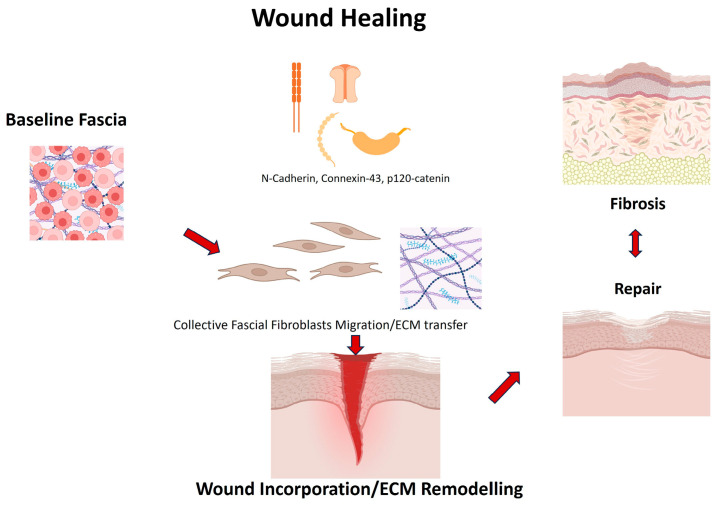
Collective fascia fibroblast migration and ECM transfer as a mechanism of repair in wound healing. Fascia fibroblasts migrate collectively toward wound sites as pre-assembled ECM patches containing vascular and immune components. This coordinate migration depends on N-cadherin, Connexin43 and p120-catenin, which regulate cell–cell adhesion, calcium oscillation synchrony and scar formation [34,37,38]. Transferred fascial ECM integrates into the wound bed, contributing to tissue repair but also potentially driving fibrosis if regulatory checkpoints are disrupted. The balance between regeneration and fibrosis define fascia’s dual role as an “active donor tissue” in wound healing.

Mechanistic refinements followed: Jiang et al. [37] identified N-cadherin as indispensable for swarming and scar formation, Wan et al. [34] showed Connexin43-mediated calcium oscillations synchronize migration, and Rahjendran et al. [38] demonstrated that silencing p120-catenin reduces scarring and ECM transfer. These studies offer causal leverage via genetic or pharmacological perturbations, yet have potential off-target effects, developmental compensation and incomplete reporting of randomization/blinding temper causal claims; importantly, dosing windows that minimize toxicity while preserving necessary tissue integrity remain undefined. Together, these data highlight molecular checkpoints of fascial regeneration, which are not mere correlates but functional regulators of repair–fibrosis balance. Importantly, they offer potential therapeutic entry points: for instance, pharmacological modulation of Cx43 or N-cadherin could redirect healing trajectories. Yet, translation to humans will require careful calibration, since excessive suppression of these pathways may compromise necessary tissue integrity. Standardized outcome measures and head-to-head comparisons across perturbations will be needed to determine effect sizes and safety margins relevant for clinical translation (Figure 2).

A second theme is the heterogeneity and plasticity of fascia progenitors. Lineage-tracing studies identified FAPs and PDGFRα+ cells as regulators of regenerative versus fibrotic outcomes [8,9], while single-cell transcriptomics revealed Prg4+ progenitors in fascia-associated compartments [39]. Such cellular diversity explains the variable outcomes observed clinically: fascia can either regenerate or fibrose depending on which progenitor subsets dominate. Human studies strengthen this concept. Chen et al. [36] demonstrated that FDSCs differ markedly from ADSCs, with higher expression of HMOX1, HIF-1α and VEGFα and superior angiogenic activity, both in vitro and in co-transplantation assays. However, the small number of human donors and reliance on in vitro and surrogate angiogenic endpoints limit external validity. Di Taranto et al. [28] further documented functional differences between superficial and deep fascia-derived ASCs, highlighting that even within fascia, niche-specific variability is critical. This anatomical resolution is a strength, though sampling was restricted to specific sites, so conclusions may not extend to other fascial compartments. These findings emphasize fascia as a unique and heterogeneous stem cell reservoir, whose regenerative quality depends on both anatomical locations and microenvironment. Prospective stratification by anatomical layer and donor characteristics will be essential to resolve context-specific effects.

Third, the review consolidates the paracrine and immunomodulatory role of fascia-derived progenitors. Wu et al. [26] showed that extracellular vesicles from hypoxia-preconditioned ASCs enhanced HIF-1α-mediated angiogenesis and flap survival in vivo, while Baptista et al. [31] and Ishiuchi et al. [32] demonstrated that fascial scaffolds promote stem cell adhesion, ECM deposition and regenerative phenotypes more effectively than nonfascial constructs. Beyond these paracrine effects, fascia is also implicated in a supportive scaffold in peripheral nerve repair. Paiva et al. [45] demonstrated in a rat model of end-to-side neurorrhaphy that the combination of fascia with platelet gel and adipose-derived stem cells significantly enhanced axonal regeneration, achieving functional recovery and nerve fiber counts comparable to controls. These studies highlight mechanistic plausibility and functional readouts (strengths), yet preconditioning protocols, scaffold composition and EV isolation methods vary widely; endpoints often emphasize histology or short-term function rather than long-term, clinically meaningful outcomes. These findings align fascia with the broader concept of stromal niches as paracrine signaling hubs, extending beyond structural support to active immune–vascular regulation. Harmonization of preconditioning and scaffold protocols would improve reproducibility and facilitate meta-analytic synthesis.

The translational implications of this biology are beginning to be explored clinically. Atilano et al. [15] demonstrated that targeting fascia directly, via subfascial PRP injections, achieved superior outcomes in greater trochanteric pain syndrome compare with tendon-focused approaches. Herold et al. [46] showed that metabolic preconditioning of adipofascial flaps with insulin and glucocorticoids improved graft survival, reinforcing the concept that fascia may also harbor pathological progenitors, as demonstrated in Dupuytren’s disease, when mesenchymal stem cells contribute to fibrotic remodeling. Notwithstanding these signals, clinical studies are few, involve small samples and often have short follow-up; PRP preparation/dosing is heterogeneous and allocation/blinding are not always reported, which increases risk of bias and may inflate effect sizes. These findings underscore the dual nature of fascia biology, capable of driving regeneration or fibrosis, depending on cellular and environmental context. Future trials should predefine core outcome sets (pain, function, imaging), standardize PRP protocols and incorporate stratification by fascial layer to enhance interpretability and translatability.

**Figure 3:**
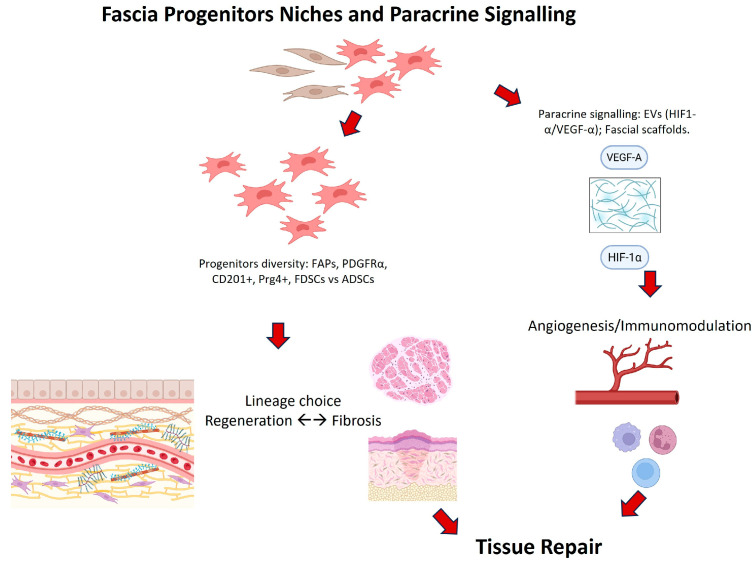
Fascia as a mechanobiological hub and stem cells reservoir driving tissue repair. Schematic representation of the main cellular and molecular mechanisms linking fascia progenitor biology to regenerative outcomes. Diverse progenitor populations contribute to the fascial regenerative niche. Through paracrine signaling, including extracellular vesicles enriched in HIF-1α and VEGF-A and the use of fascial scaffolds, these cells promote angiogenesis and immunomodulation. Lineage decisions between regeneration and fibrosis determine the final outcome of fascia-mediated tissue repair.

Conceptual reviews [16,48,49] provide a theoretical framework that resonates with these empirical findings, positioning fascia as both a mechanosensitive organ and the dominant ECM source in wound repair. By synthesizing mechanistic, translational and conceptual evidence, this review highlights fascia as a bridge between stem cell biology, mechanobiology and regenerative medicine (Figure 3).

Significant limitations must be acknowledged. Most data were derived from in vitro and small-animal models with inherent interspecies and scaling limitations. Clinical studies remain few, underpowered and methodologically heterogenous, limiting external validity. Anatomical bias toward superficial fascia leaves various topographical fasciae unexplored, despite their potential clinical relevance. Furthermore, the pathways identified as regenerative regulators (N-cadherin, connexin43, p120-catenin) may not yet be targetable in humans, raising concerns about translatability. Heterogenous cell isolation, culture conditions and outcome definitions further hinder cross-study comparison; single-cell analyses are susceptible to batch and dissociation artifacts; and selective reporting/publication bias cannot be excluded. These limitations collectively reduce the certainty of effect estimates and may overstate coherence across models; consequently, our conclusions regarding clinical relevance should be interpreted as hypothesis-generating rather than definitive.

Beyond these considerations, recent methodological advances are expanding the experimental landscape of fascial biology. Single-cell and spatial transcriptomic analyses have revealed the heterogeneity of fascial fibroblast [50] and progenitor populations, delineating PDGFRα, CD201+ and Prg4+ subsets within distinct compartments [39,40]. Organoid and organ-on-chip models have begun to simulate the fascial microenvironment, reproducing its mechano-biochemical properties and enabling dynamic observation of progenitor behavior under defined tension or hypoxia [43,44]. In parallel, advances in biomaterials, particularly biomimetic scaffolds composed of aligned collagen fibers, electrospun nanofibers or decellularized fascial matrices, are emerging as promising platforms for guided cell migration and regenerative integration [31,32]. While still in early development, these technologies are expected to bridge the gap between descriptive fascial anatomy and translational tissue engineering.

Moving forward, future research should prioritize several directions. First, large-scale, multicenter randomized controlled trials are needed to evaluate fascia-target interventions such as PRP injections, fascia-derived cell therapies and biomimetic scaffolds with standardized outcomes (pain, function, imaging). Second, systematic exploration of various types of fasciae could expand the therapeutic scope. Third, advanced mechanistic studies combining single-cell transcriptomics, live imaging and biomechanical modeling should dissect mechanoresponsive signaling in human fascia. Fourth, comparative analyses of FDSCs versus ADSCs or bone marrow MSCs will clarify whether fascia is indeed a superior progenitor source. Fifth, at preclinical level, standardized models that clearly define fascial sublayers are needed to disentangle the specific contributions of different fasciae. Advanced lineage-tracing tools and single-cell multi-omics will help fascia’s progenitor hierarchies, while biomechanical bioreactors and organ-on-chip models could replicate in vivo fascial dynamics more accurately. Finally, biomaterial and pharmacological strategies should aim to translate the molecular checkpoints identified in animal models into clinically applicable therapies. Translating fascia biology into clinical practice will require overcoming several challenges. First, mechanistic targets identified in animal models (such as N-cadherin, Connexin43 and p120-catenin) must be validated in human tissues using standardized assays and multicenter collaborations. Second, heterogeneity in fascial anatomy, sampling methods and outcome definitions must be minimized through consensus frameworks and shared biobanking resources. Third, integrating biomechanical modeling, single-cell multi-omics and longitudinal imaging will be essential to track fascia remodeling dynamically in patients. Finally, developing reproducible manufacturing standards for fascia-derived cell products and bioengineered scaffolds will be crucial to ensure safety, regulatory compliance, and scalability. Together, these strategies could substantially accelerate the clinical translation of fascia-targeted therapies. Across these directions, rigorous design features (preregistration, randomization, blinding, adequately powered samples and long-term follow-up) and harmonized definitions of fascial layers and interventions will be critical to strengthen inference and mitigate bias.

## 5. Conclusions

In conclusion, fascia emerges from this systematic review as a dynamic and multifunctional tissue, a stem cell niche, a mechanosensitive regulator and the principal ECM source of repair. While current clinical data remain preliminary and limited by small, non-randomized designs with variable risk of bias, the convergence of mechanistic and preclinical findings provides a rational for further investigation of fascia as a potential therapeutic target, rather than evidence of established clinical effectiveness. However, given the limitations discussed, clinical claims should remain provisional until validated by well-designed, adequately powered human studies with standardized protocols and outcomes.

Bridging the translational gap with high-quality clinical studies and extending research beyond superficial fascia will be essential to unlock the full regenerative promise of this tissue.

## Figures and Tables

**Table 1:** Summary of included in vitro studies investigating fascia-derived stem/progenitor cells. The table reports study design, cell source, key mechanobiological findings and assessed regenerative or fibrotic outcomes.

Authors	Fascia Type	Cell Type	Primary Outcomes	Key Findings
Hung et al., 2014 [20]	Pelvic fascia	ASCs	Collagen I/III, elastin	ASCs enhanced ECM deposition and scaffold remodeling
Zhang et al., 2020 [21]	Subcutaneous fascia	Fascia adipocytes	Morphology, inflammatory profile	Fascia adipocytes identified as a distinct, pro-inflammatory, low-lipid cell type
Su et al., 2014 [22]	Fascial scaffold	Endometrial MSCs	Differentiation, ECM	Differentiated into fibroblastic and smooth muscle phenotypes producing ECM
Ayala et al., 2015 [23]	Abdominal fascia	ASCs	Vascularization, biomechanics	Composite fascia improved angiogenesis and tensile strength
Lo et al., 2024 [24]	Pelvic fascia	Amniotic fluid SCs	Tensile properties	Scaffold seeded with AFSCs enhanced tensile resistance and biocompatibility
Hindocha et al., 2011 [25]	Palmar fascia	Resident stem cells	Cell identification	Resident stem cells identified in Dupuytren’s fascia
Wu et al., 2022 [26]	Fascial flap	ASC-derived EVs	Angiogenesis, flap survival	EVs improved HIF-1α signaling and flap survival
Cho et al., 2021 [27]	Fascial repair model	MSCs + GFs	ECM deposition, healing	GF-loaded scaffolds improved fascial healing in vitro
Di Taranto et al., 2015 [28]	Subcutaneous fascia	ASCs	Stemness, differentiation	Significant heterogeneity between superficial and deep fascia ASCs
Iqbal et al., 2014 [29]	Palmar fascia	MSCs	Stem cell identification	First identification of resident/circulating MSCs in Dupuytren’s fascia
Roman et al., 2016 [30]	Pelvic fascia	MSCs	Biocompatibility, scaffold design	Scaffold mimicked fascia mechanics and supported cell growth
Baptista et al., 2021 [31]	Fascial constructs	MSCs	Adhesion, ECM phenotype	Microenvironment promoted adhesion and regenerative phenotypes
Ishiuchi et al., 2023 [32]	Fascial scaffolds	MSCs	ECM deposition	MSCs in fascial scaffolds deposited more ECM than dermal constructs
Zhang et al., 2018 [33]	Thoracolumbar fascia	Fibroblasts	Histology, mechanics	Described collagen organization, cellularity, and mechanical properties
Wan et al., 2021 [34]	Subcutaneous fascia (back skin, mouse and human)	Subcutaneous fascia (back skin, mouse and human)	Cx43 expression, calcium oscillations, fascia fibroblast migration, matrix mobilization, scar size and composition	Cx43 is upregulated in fascia EPFs after injury; gap junction communication sustains collective migration and fascia matrix mobilization into wounds; inhibition of Cx43 (2-APB, GAP27) or calcium signaling reduces fascia mobilization and scar formation.
Correa-Gallegos et al., 2023 [35]	Subcutaneous fascia	CD201+ progenitors	Lineage tracing, fibroblast transition	CD201+ progenitors sequentially generate fibroblasts and myofibroblasts
Chen et al., 2025 [36]	Human superficial fascia	Fascia-derived stem cells (FDSC)	Transcriptomics, angiogenesis, graft retention	FDSCs expressed higher HMOX1, HIF-1α, and VEGFa; promoted angiogenesis and improved graft retention

**Table 2:** Summary of animal studies.

Authors	Fascia Type	Cell Type	Primary Outcomes	Key Findings
Correa-Gallegos et al. [10]	Subcutaneous fascia	Fibroblasts	Wound closure, ECM	Fascia fibroblasts mobilize as pre-assembled ECM patches
Jiang et al. [37]	Subcutaneous fascia	Fibroblasts	Scar size, migration	N-cadherin essential for collective migration
Wan et al. [34]	Subcutaneous fascia	Fibroblasts	Migration, calcium signaling	Connexin43 regulates collective migration
Rajendran et al. [38]	Subcutaneous fascia	Fibroblasts	ECM transfer, scarring	p120 controls supracellular organization in fascia fibroblasts
Correa-Gallegos et al. [35]	Subcutaneous fascia	CD201+ progenitors	Lineage tracing	CD201+ cells sequentially generate fibroblasts/myofibroblasts
Joe et al. [8]	Muscle fascia	FAPs	Myogenesis, fibrosis	FAPs aid myogenesis but drive fibrosis if dysregulated
Uezumi et al. [9]	Muscle fascia	PDGFRα+ progenitors	Adipogenesis	PDGFRα+ progenitors cause ectopic fat
Almet et al. [39]	Subcutaneous/deep fascia in murine back skin	Fascia Fibroblasts	scRNA-seq, spatial transcriptomics	Identified dynamic fibroblast states during wound healing; fascia fibroblasts contribute to transient upper and lower scar compartments
Camargo et al. [40]	Murine fascia	ASCs	Viability	HBOT + ASCs improved fascial survival
Wu S. et al. [26]	Fascial flap	ASC-derived EVs	Angiogenesis, viability	EVs enhanced HIF-1α and vascularization
Marie et al. [41]	Fascial tissue	MSCs	Elasticity, mechanics	MSCs improved fascial biomechanics
Dai et al. [42]	Fascial environment	MSCs	Integration	Better integration in fascia-rich sites
Tavakkoli Tabassi et al. [43]	Fascial scaffolds	MSCs	Repair outcomes	MSCs + PRP improved fascial integration
Lee et al. [44]	Umbilical fascia	Endothelial-like	Anatomy	Identified novel vascular structures with fascia
Hindocha S. et al. [25]	Palmar fascia	Progenitors	Stemness	Progenitors identified in diseased fascia
Di Taranto et al. [28]	Subcutaneous fascia	ASCs	Stemness	Regional ASC heterogeneity in fascia
Pavia et al. [45]	Muscle fascia (autologous, used to wrap neurorrhaphy)	Adipose-derived mesenchymal stem cells (autologous, uncultured)	Peripheral nerve regeneration, muscle reinnervation, functional recovery (peroneal functional index)	ESN wrapped with fascia and platelet gel carrying ADSCs improved functional recovery and nerve fiber counts, reaching values comparable to controls; fascia served as scaffold to retain stem cells and support regeneration

**Table 3:** Risk of bias and applicability concerns summary of in vitro studies.

Authors	D1 Cell Source Characterization	D2 Biological Replicates	D3 Controls	D4 Blinded Assessment	D5 Data Completeness	D6 Statistics	D7 Outcome Validity
Hung et al. [20]	High	Unclear	High	Low	High	Unclear	Low
Zhang et al. [21]	High	Unclear	High	Low	High	High	Unclear
Su et al. [22]	High	Unclear	High	Low	High	High	Unclear
Ayala et al. [23]	High	Unclear	High	Low	High	Unclear	Unclear
Lo et al. [24]	High	Unclear	High	Low	High	High	Unclear
Hindocha et al. [25]	High	Unclear	High	Low	High	High	Unclear
Wu et al. [26]	High	Unclear	High	Low	High	High	Unclear
Cho et al. [27]	High	Unclear	High	Low	High	High	High
Di Taranto et al. [28]	High	High	High	Low	High	High	High
Iqbal et al. [29]	High	Unclear	High	Low	High	High	Unclear
Roman et al. [30]	High	Unclear	High	Low	High	High	Unclear
Baptista et al. [31]	High	High	High	Low	High	High	High
Ishiuchi et al. [32]	High	High	High	Low	High	High	High
Zhang et al. [33]	Low	Unclear	Unclear	Unclear	Unclear	Unclear	Unclear
Wan et al. [34]	High	Unclear	High	Low	High	High	Unclear
Correa-Gallegos et al. [35]	High	Unclear	High	Low	High	High	High
Chen et al. [36]	High	High	High	Low	High	High	High

**Table 4:** Risk of bias and applicability concerns summary about animal studies.

Authors	D1	D2	D3	D4	D5	D6	D7	D8	D9	D10	D11	D12	D13	D14
Correa-Gallegos et al. [10]	Unclear	Unclear	Low	Unclear	Unclear	Unclear	Low	Low	Low	Low	Low	Low	Low	Some concerns
Jiang et al. [37]	Unclear	Unclear	Low	Unclear	Unclear	Unclear	Unclear	Unclear	Unclear	Unclear	Low	Unclear	Unclear	Some concerns
Wan et al. [34]	Unclear	Unclear	Low	Unclear	Unclear	Unclear	Unclear	Unclear	Unclear	Unclear	Low	Unclear	Unclear	Some concerns
Rajendran et al. [38]	Unclear	Unclear	Low	Unclear	Unclear	Unclear	Unclear	Unclear	Unclear	Unclear	Low	Unclear	Unclear	Some concerns
Correa-Gallegos et al. [35]	High	Low	High	Unclear	Low	Low	High	Low	Low	High	High	Low	High	Some concerns
Joe et al. [8]	Unclear	Low	High	Unclear	Low	Low	High	Low	Some concerns	Unclear	Low	Low	High	Some concerns
Uezumi et al. [9]	Unclear	Low	High	Unclear	Low	Low	High	Low	Some concerns	Unclear	Low	Low	High	Some concerns
Almet et al. [39]	Low	Low	Unclear	Low	Low	Low	High	Low	Some concerns	Low	Unclear	Low	High	Some concerns
Camargo et al. [40]	High	Unclear	High	Unclear	Low	Unclear	High	Low	Low	High	Low	Unclear	High	Some concerns
Wu S. et al. [26]	Unclear	Unclear	Low	Unclear	Unclear	Unclear	Unclear	Unclear	Unclear	Unclear	Unclear	Unclear	Unclear	Some concerns
Marie et al. [41]	Unclear	Low	High	Unclear	Low	Low	High	Some concerns	High	Unclear	High	Low	High	Some concerns
Dai et al. [42]	Unclear	Low	High	Unclear	Low	Low	High	Some concerns	Low	High	Low	Low	High	Some concerns
Tavakkoli Tabassi et al. [43]	Unclear	Low	Unclear	NA	Low	Low	High	Some concerns	High	Unclear	Low	High	High	Some concerns
Lee et al. [44]	Low	Low	High	NA	NA	Low	High	Some concerns	High	Low	Low	Low	High	Some concerns
Hindocha S. et al. [25]	Unclear	Unclear	Low	Unclear	Unclear	Unclear	Unclear	Unclear	Unclear	Unclear	Unclear	Unclear	Unclear	Some concerns
Di Taranto et al. [28]	Unclear	Unclear	Low	Unclear	Unclear	Unclear	Unclear	Unclear	Unclear	Unclear	Unclear	Unclear	Unclear	Some concerns
Pavia et al. [45]	High	Unclear	High	Unclear	Low	Unclear	High	Low	Low	High	Low	Unclear	High	Some concerns

D1: sequence generation; D2: allocation concealment; D3: baseline similarity; D4: random housing; D5: blinding caregivers; D6: blinding assessors; D7: incomplete data; D8: selective reporting; D9: other bias; D10: dosimetry; D11: mechanobiology control; D12: sample size calc; D13: welfare; D14: external validity.

**Table 5:** Risk of bias and applicability concerns summary about clinical studies.

Authors	D1	D2	D3	D4	D5	D6	D7	D8	D9	D10	D11	D12	Overall RoB
Atilano L. et al. [15]	Low	Low	Low	Low	Moderate	Low	Low	Low	Low	Moderate	Low	Low	Low
Herold C. et al. [46]	Low	Low	Low	Low	Moderate	Low	Low	Low	Low	Moderate	Low	Low	Low
Hindocha S. et al. [25]	Moderate	Moderate	Low	Low	Low	Moderate	High	NA	NA	Moderate	High	Moderate	High
Iqbal SA et al. [47]	Moderate	Moderate	Moderate	Moderate	Moderate	Moderate	Moderate	Moderate	Moderate	Moderate	Moderate	Moderate	Serious

D1: confounding; D2: selection of participants; D3: classification of interventions; D4: deviations from interventions; D5: missing data; D6: measurement of outcomes; D7: selection of reported results; D8: sample size and precision; D9: follow up; D10: co-interventions; D11: standardization of assessments; D12: protocol or registration; overall RoB.

## Data Availability

No new data were created or analyzed in this study.

## Abbreviations

The following abbreviations are used in this manuscript:

ECM	Extracellular matrix
FAPs	Fibro-adipogenic progenitors
MSCs	Mesenchymal stem cells
ASCs	Adipose-derived stem cells
